# Chlorophyll-a Estimation Around the Antarctica Peninsula Using Satellite Algorithms: Hints from Field Water Leaving Reflectance

**DOI:** 10.3390/s16122075

**Published:** 2016-12-07

**Authors:** Chen Zeng, Huiping Xu, Andrew M. Fischer

**Affiliations:** 1 School of Ocean and Earth Science, Tongji University, Shanghai 200092, China; xuhp@sidsse.ac.cn; 2 Department of Earth, Ocean and Atmospheric Sciences, University of British Columbia, Vancouver, BC V6T 1Z4, Canada; 3 Institute of Deep-Sea Science and Engineering, Chinese Academy of Sciences, Sanya 572000, China; 4 Institute for Marine and Antarctic Studies, University of Tasmania, Launceston 7250, Australia; andy.fischer@utas.edu.au

**Keywords:** water leaving reflectance, skylight downwelling radiance, chlorophyll-a estimation, MODIS, VIIRS

## Abstract

Ocean color remote sensing significantly contributes to our understanding of phytoplankton distribution and abundance and primary productivity in the Southern Ocean (SO). However, the current SO in situ optical database is still insufficient and unevenly distributed. This limits the ability to produce robust and accurate measurements of satellite-based chlorophyll. Based on data collected on cruises around the Antarctica Peninsula (AP) on January 2014 and 2016, this research intends to enhance our knowledge of SO water and atmospheric optical characteristics and address satellite algorithm deficiency of ocean color products. We collected high resolution in situ water leaving reflectance (±1 nm band resolution), simultaneous in situ chlorophyll-a concentrations and satellite (MODIS and VIIRS) water leaving reflectance. Field samples show that clouds have a great impact on the visible green bands and are difficult to detect because NASA protocols apply the NIR band as a cloud contamination threshold. When compared to global case I water, water around the AP has lower water leaving reflectance and a narrower blue-green band ratio, which explains chlorophyll-a underestimation in high chlorophyll-a regions and overestimation in low chlorophyll-a regions. VIIRS shows higher spatial coverage and detection accuracy than MODIS. After coefficient improvement, VIIRS is able to predict chlorophyll a with 53% accuracy.

## 1. Introduction

The Southern Ocean (SO) contains relatively high seasonal levels of phytoplankton biomass in its coastal waters due to the complex processes of ice melt and intense seasonal light availability. Seasonal blooms in this region play a significant role in driving global biogeochemistry cycling [1,2]. Satellite imagery can provide high spatial and temporal coverage of the SO in the southern spring and summer (from October to March), and is useful for understanding the patterns and variability of phytoplankton biomass across broader temporal and spatial scales. This is an advantage over in situ environmental detection [3,4], where severe weather can make routine and comprehensive sampling difficult.

However, imperfect atmospheric correction of satellite images over the SO region produces uncertainty in chlorophyll-a estimation [5,6,7]. For example, the OC2v2 algorithm, when applied to CZSC and SeaWiFS imagery, underestimated chlorophyll-a (0.7–43 mg/m^3^) concentrations by 60% in the west marginal sea of the Antarctic Peninsula [8]. A potential reason for this is that the SO water-leaving reflectance (Rrs) is high in the blue band (443 nm) and low in green band (555 nm), when compared with the global open ocean [9]. An underestimation of 87% was also found in the chlorophyll concentration range of 0–1.0 mg/m^3^ and by 30% in concentration values of >5 mg/m^3^ in the South Georgia area (54.5° S, 37° W) [10]. A strong relationship was reported between inversion and in situ chlorophyll-a estimates, however, underestimation still existed for chlorophyll concentrations between 0.1 and 1.5 mg/m^3^ in the Ross Sea [4]. The OC3 algorithm coefficients were improved with in situ samples and provided better agreement (*r*^2^ = 0.64) near the Antarctica Peninsula [11]. Since all inversions above have their own scope and regional limitation, SO chlorophyll-a inversion accuracy deserves improvement to enhance chla estimation.

The aim of this study is to assess the accuracy of satellite-based chlorophyll-a estimation within coastal Antarctic waters. Through the collection of hyper-spectral downwelling irradiance and water leaving reflectance (Rrs) around the West Antarctica Peninsula (WAP), an area which lacks a sufficient in situ validation datasets for satellite remote sensing, we evaluate the chlorophyll-a algorithm estimations from three aspects: first, the downwelling irradiance total signal was collected just above the sea surface and was used to derive atmospheric spectral information such as particle scattering and gas and aerosol absorption; second, the hyper-spectral Rrs (±1 nm) were used to determine detailed bio-optical characteristics of WAP waters; finally, our data were collected after launching of VIIRS. For this new sensor, reflectance validation samples are seldom available from SeaBASS [12]. This manuscript makes a comparison between MODIS and VIIRS performance using current OC satellite chlorophyll-a algorithms and improves regional chlorophyll-a estimation coefficients of OC algorithms.

## 2. Materials and Methods

### 2.1. Above Water Measurement and Processing

**Figure 1:**
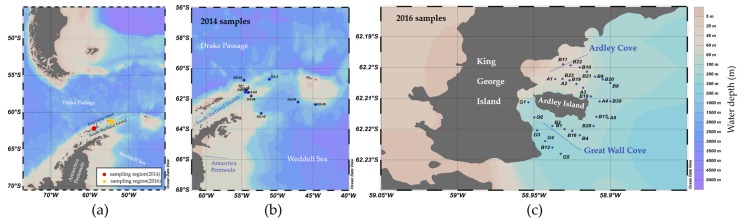
(**a**) Map of sample distribution in SO around Antarctic Peninsula with topography from ETOPO1 [16]; (**b**) Map of detail sample distribution during 10 February to 19 February 2014; (**c**) Map of detail sample distribution during 6 January to 27 January 2016.

Our research measured nine water leaving reflectance samples between 10 and 19 February 2014 (Figure 1b) and 30 water leaving reflectance (Rrs) samples around AP between 6 and 27 January 2016 (Figure 1c). An ASD Fieldspec HandHeld 2 was used to obtain water leaving reflectance with high band resolution of ±1 nm and a spectral range from 325 to 1075 nm, containing visible and infrared bands. The device had conducted band peak shifting calibration, standard plaque reflectance calibration, and sensor radiance calibration before and after cruises.

Hyper-spectral downwelling irradiance and water leaving reflectance were based on above-water measurements using the methods of Mueller et al. [13]. Compared with under water measurements, above-water measurement provide many advantages. First, they are of higher spectral resolution (±1 nm band resolution) as underwater devices have limited spectral bands (profiling reflectance radiometer has six upwelling and downwelling irradiance in 412, 443, 490, 555, and 656 nm). Underwater measurements also need to be extrapolated to surface values, a process which usually introduces uncertainty [14]. Second, above water leaving reflectance can be directly compared to satellite derived normalized water-leaving (nLw) radiance. Third, underwater measurements are also subject to air water interface transformation [15], which varies when light incident angle changes.

Simultaneous meteorological parameters such as sea ice location, sky clarity index, and wind speed were also recorded. Overcast weather appeared during almost the whole sampling period. The homogeneous light condition from overcast weather produced a uniform skylight radiance condition more conducive for Rrs measurements than cloudy conditions or intense glare [14]. Under cloudy conditions and intense glare, water receives heterogeneous and patchy irradiance and reflects a variety of light intensity in different scattering angles. This creates difficulties for atmospheric correction and can lead to uncertainties in Rrs detection.

According to the ocean color protocol [13], we sampled above-water Rrs within the recommended zenith (θ) and azimuth (Φ) angles at 40° and 135°. A fishing rod of 3 m length was held to avoid shipboard interference. We collected 15 samples of water, skylight and standard plague measurements at each sampling location. Every sample had 2 duplicates. For each sample, we chose a minimum of 10 signals and averaged them to get water, skylight and standard plaque signal to decrease the signal noise for each sample.

Because the air-water interface ratio (r) changes with wind speed, we followed the methods of [17] to computer r (see Equation (1)). To improve the atmospheric correction, we used 936 nm as the infrared black pixel. The r value in this manuscript from AP data is 4.4 ± 1.1 (%). Then, Equation (2) [18] was applied to obtain water-leaving reflectance from respective water, skylight and standard plaque signal. Finally, all bands were offset by the value at 936 nm:

(1) $$r=\frac{S_{water\left(\lambda_{936}\right)}-0.015\times S_{sky}\left(\lambda_{936}\right)}{S_{sky}\left(\lambda_{936}\right)}$$

(2) $$R_{rs}=\frac{[S_{water}-rS_{sky}]\times \rho_{p}}{\pi \times S_{p}}$$

where, r is air-water interface reflectance rate (%) [13], Rrs is water-leaving reflectance (sr^−1^), $S_{water}$, $S_{sky}$, $S_{p}$ is the digital signal value measured for water, skylight and standard plaque radiance, $\rho_{p}$ is the standard plaque bi-directional reflectance (99% for this study) and $\lambda_{936}$ is band wavelength 936 nm. High bi-directional reflectance (99%) provided a high signal-to-noise ratio (SNR) for the downwelling irradiance signal [14]. This process eliminates atmosphere interference and provides a normalized Rrs.

### 2.2. In Situ Chlorophyll-a Measurements

Samples for chlorophyll-a concentration were filtered onto 47 mm glass fiber filters (GF/F) using low vacuum pressure (<10 mm Hg), wrapped with aluminum foil and immediately stored at −80 °C until analysis within two months. Each sample was collected in duplicate with 500 mL water for each. Then, chlorophyll-a was extracted by 90% acetone overnight and centrifuged for 10 min. Finally, its supernatant was analyzed in a 10AU™ Field and Laboratory Fluorometer for total chlorophyll-a. After adding 10% HCl and settling for 1 min, the pheophytin concentration was also collected. Chlorophyll-a concentration was calculated by the chlorophyll-a standard curve. The chlorophyll-a standard curve was regressed with the chlorophyll-a standard product from Sigma Aldrich across various concentration gradients. All analyses were conducted under dim light.

### 2.3. Spectrum Sensitivity Analysis

To determine the best band for satellite chlorophyll-a estimation, we applied Gitelson et al.’s [19] algorithm on spectrum sensitivity. For band-ratio algorithm:

(3) $$chlorophyll\;\alpha \;\sim \;\frac{blue\;band}{green\;band}$$

Using 488 nm as the blue band and 400 nm as the green band we calculated the ratio of 488 nm/400 nm. We then applied least-squares method to approximate the linear-regression coefficients between the ratio values from all of the field Rrs samples and in situ chlorophyll-a (*n* = 39), and estimated the residual error. Then we moved the green band choice one by one, till we reached the 700 nm band wavelength. When plotting all of the estimated residual error for different green band selections, we will get the optimal band for green band chlorophyll-a estimation. Next we confirmed green band at the optimal green band choice and applied above method to traverse all of 400–700 nm to find the optimal blue band.

### 2.4. Satellite and In Situ Derived Data Matching

For matching pairs between in situ and satellite data, we collected all images from visible bands in MODIS AQUA/TERRA (443, 469, 488, 531, 555, 645, 667, 678 nm) and VIIRS (410, 443, 486, 551, 671 nm) during the cruises. These daily L3 water leaving reflectance products have a spatial resolution of 4.25 km and have been georeferenced and atmospherically corrected. These products can be directly compared with our in situ reflectance water measurements. Due to overcast weather during almost all of the sampling period, we chose the following thresholds for matching in situ and satellite pairs, (1) interval time between in situ and satellite derived samples was no more than 72 h [7,20]; and (2) satellite 3 × 3 grid pixel window were averaged at the center of in situ sample location to lower data noise and cloud interference [21,22]. This temporal threshold won’t decrease the accuracy, because Johnson et al. [20] found SO multi-day composite products (8 day) still provided good agreement with in situ samples.

### 2.5. MODTRAN-Based Atmospheric Downwelling Radiance Simulation

The above-water methodology [13] and ocean color protocol applies the same rule to subtract atmospheric signal from total water-leaving radiance. While above-water methodology measures skylight downwelling radiance directly and then subtracts it from total water-leaving radiance, satellite correction applies pre-calculated atmospheric radiation transfer models to simulate atmospheric signals, where its aerosol absorption is estimated by sensors’ near infrared bands. Thus we compared field skylight downwelling radiance and MODTRAN simulated total radiance to study the deficiencies of the current atmospheric simulation around AP.

MODTRAN (version 5) was used here and major options were: MODTRAN scaled discrete ordinate radiance transfer, H_2_O component at 0.4, 1 g/cm^3^, marine aerosol (default visibility 23 km), nimbostratus cloud layer, wind speed 7.74 m/s. Path type: 40° sun zenith angle and 120° sun azimuth angle according to field measurements. Normalization process (percentage (%) = ($S_{skylight}$ (λ))/(max($S_{skylight}$)) × 100%, where λ is band number) was then performed to eliminate environmental effect on individual samples.

## 3. Results

### 3.1. Skylight Downwelling Radiance

Total downwelling radiance is the incident contribution reaching sea surface, which is a summation of extraterrestrial downwelling irradiance, Rayleigh scattering, gas and aerosol absorption [23,24]. The rule of atmospheric correction is to remove all those contributions from atmosphere, leaving only water-leaving radiance for the derivation of an ocean color product. Atmospheric correction is important for water-leaving reflectance because the atmosphere comprises over 90% of the total water leaving signal [25].

**Figure 2:**
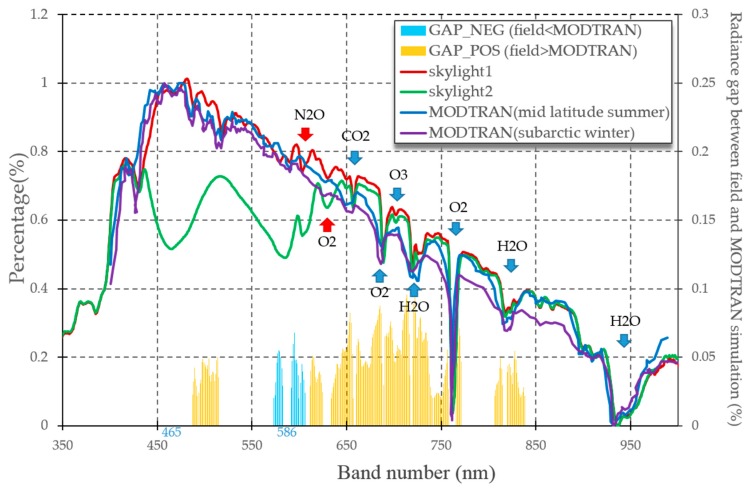
Skylight downwelling radiance distribution from AP and MODTRAN simulation. The histogram is the skylight1 subtracting from the MODTRAN Mid-Latitude summer simulation.

No obvious gaps appear in the mid-latitude summer atmosphere and high-latitude winter atmosphere simulations, both showing a similar tendency to decrease from around 465 nm (Figure 2). However, the high-latitude summer model values are about 3.99% lower than mid-latitude summer model during 600–900 nm band interval. Those two MODTRAN simulation are the models with greatest gap between all six atmospheric models (Tropical Atmosphere, Mid-Latitude Summer, Mid-Latitude Winter, Sub-Arctic Summer, Sub-Arctic Winter, 1976 US Standard Atmosphere).

Skylight 1 & 2 are common samples for skylight spectra collected in AP. Skylight 1 shows great agreement with MODTRAN simulations, especially with the Mid-Latitude Summer model. Mid-Latitude summer model appears to be the optimal model for AP total downwelling radiance simulation. Their near infrared bands are quite similar, and identical absorption peaks are located at 690, 720, 760, 810 and 930 nm due to the atmospheric gas window [26]. Most of their gaps are less than 5%. Improvement is still needed for N_2_O (604 nm) and O_2_ (630 nm) gas absorption windows to better simulate AP total radiance (red arrows). Skylight 2 appears quite different from the above three (Skylight 1 and 2 MODTRAN simulations). According to field tests, those two obvious absorption peaks during 430–650 nm band intervals were caused by cumulus clouds.

### 3.2. Water Leaving Reflectance Characteristics

**Figure 3:**
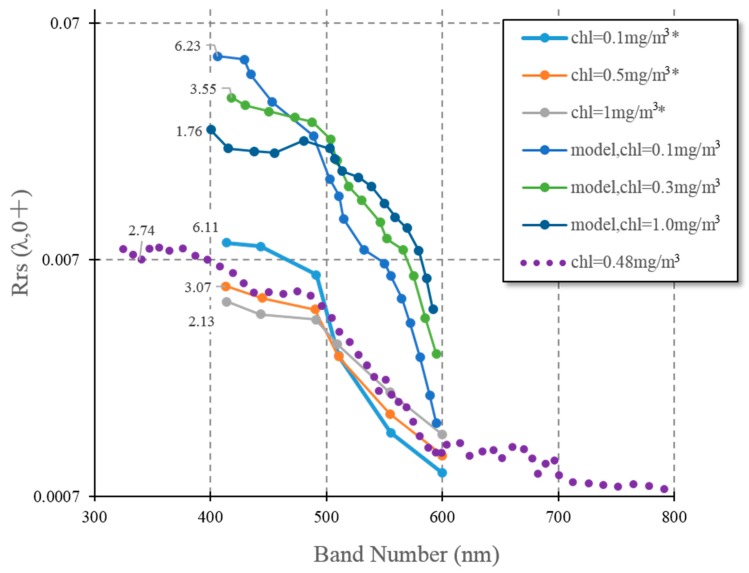
Water leaving reflectance spectrums around AP. The dot line is from our measurements, the lower three dash dot lines are in situ water spectrums in AP from Dierssen and Smith [8], and the other three dash dot lines with asterisk in legend are global water spectrum models from Morel and Maritorena [15]. The value ahead of each line is blue-green band ratio, max(Rrs(blue,443), Rrs(blue,488))/Rrs(green,555).

All of our water leaving reflectance (Rrs) samples have identical distributions and their duplicates have a standard error of less than 2.2 ± 1.8 × 10^−4^ sr^−1^. Examples are shown in Figure 3, at chlorophyll-a concentrations <1 mg/m^3^. The Rrs samples we collected and show the same tendency and range as those by Dierssen and Smith’s [8] samples collected from AP as well. The value of 0.48 mg/m^3^ chlorophyll-a sample matches Dierssen and Smith 0.5 mg/m^3^ chlorophyll-a sample of Rrs. All AP Rrs (chlorophyll-a < 1 mg/m^3^) in Figure 3 range from 0.0009 to 0.0082 sr^−1^. A turning point appears at about 490 nm. Their blue band values are high and steady between 400 and 490 nm. Water-leaving reflectance between 495–570 nm and 570–590 nm decrease steeply, followed by smooth and low water-leaving reflectance in >600 nm.

Global case I Rrs models from Morel and Maritorena [15] show greater values than those found in the AP, with ranges between 0.0014 and 0.05 sr^−1^ (chlorophyll-a <1 mg/m^3^ water). These differences highlight the peculiar optical features from AP waters. However, they share same turning point at about 490–500 nm followed by a rapid decrease in values for lower chlorophyll-a after that point. Before this turning point, reflectance with lower chlorophyll-a have higher values. It is noticed that AP water spectrums decline slowly and is smoother than global open ocean water due to its low Rrs value.

The blue-green band ratios computed by max(Rrs(blue,443), Rrs(blue,488))/Rrs(green,555) decrease with rising chlorophyll-a value in both global case I and AP waters. Global ocean waters have greater band ratio gaps between 0.1 and 1.0 mg/m^3^ chlorophyll-a range, meaning that band ratio variances become smaller in AP than in global ocean water. It is noted that the band ratio for our sample is 2.74 at 0.48 mg/m^3^ chlorophyll-a, which differs slightly from Dierssen and Smith’s [8] band ratio 3.07 at 0.5 mg/m^3^ chlorophyll. The OC3 algorithm shows that this gap equals ~6.3% per chlorophyll-a unit, which is just a small uncertainty in chlorophyll-a evaluation. This error is probably caused by different methods and processing procedures.

### 3.3. In Situ and Satellite Water Leaving Reflectance

We matched in situ and satellite data successfully obtaining 24 matching pairs for MODIS and 30 for VIIRS. The reason we choose 3 × 3 pixels and 72 h window are listed as follows. Water leaving reflectance (Rrs) signals are scattered light from the upper ocean layer, which means Rrs only contains information from the oceanic euphotic layer after ideal atmosphere information is removed. Rossby waves have higher temporal and spatial scales than window of 12 km × 12 km and 72 h, so this window selection won’t be impacted by Rossby wave. 

Besides, water around AP, controlled by phytoplankton, shows seasonal patterns due to light, nutrient and sea ice distribution [11,27]. Therefore, the ocean current field and phytoplankton concentration has greater temporal and spatial scale than this window and will remain uniform under this limitation. The data in our research also shows that the spatial standard error in the window is less than ~5.64% (calculated from the data in Table 1), and the temporal standard error of 72 h are less than ~8.26% (calculated from all remote sensing data during October 2013 and March 2014 at the same location as our samples), which supports our selection of this spatial and temporal window. More VIIRS matching pairs (n = 30) were extracted than MODIS, illustrating thehigher spatial coverage and more effective detection performance of VIIRS. Daily coverage rates showed that VIIRS measured more SO surface ocean area than MODIS by about 1.1% due to its lower SNR and greater spectral range than MODIS [28]. High SNR makes the satellite signal susceptible to information loss and more vulnerable to atmosphere gases and aerosols [28].

The average standard deviation between in situ and satellite derived Rrs is lower in MODIS (0.0076) than in VIIRS (0.0083). MODIS also has high spectral resolution band settings in visible wavelengths, with 10 bands. VIIRS only has six bands. However, the correlation coefficients are much higher from VIIRS (average of 74.522%) than from MODIS (highest coefficients are 42.10%), which can have significant contributions on chlorophyll-a estimation.

**Figure 4:**
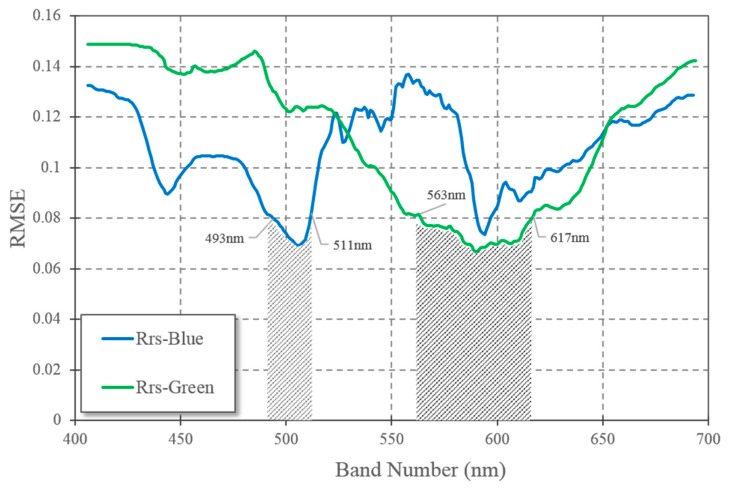
Blue-green band ratio model sensitivity on chlorophyll-a estimation, calculated from estimated residual error.

The current global OC3 algorithm for satellite chlorophyll-a estimation is built on the empirical relationship between chlorophyll-a concentration and blue-green band ratio. The sensitivity result (Figure 4) reveals that the appropriate blue band choice for SO is the 493–511 nm interval, which is higher than global case I waters, max(443, 488) nm band. The optimal band for green band in SO is between 564 and 617 nm, which is also higher than global water green band 555 nm. Therefore, no big advantage appears in either current satellite setting 486/555 nm band in VIIRS or 488/555 nm band in MODIS, both providing similar sensitivity for chlorophyll-a estimation.

**Figure 5:**
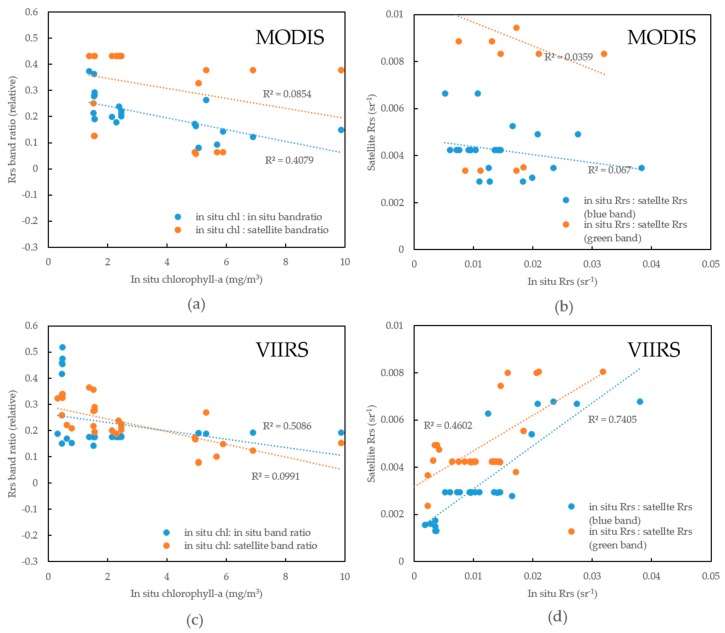

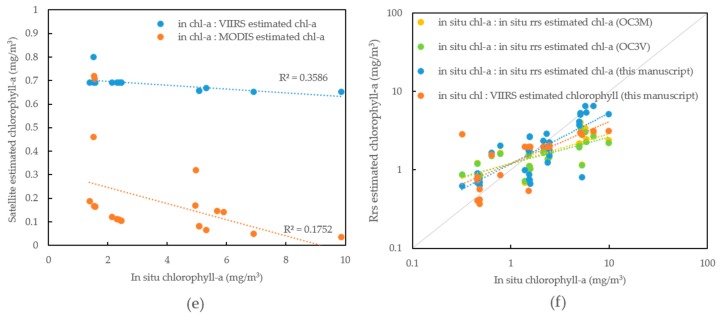
(**a**) Correlations between Rrs band ratio and in situ chlorophyll-a for MODIS matching pairs and its corresponding in situ Rrs; (**b**) Correlations between MODIS and in situ blue and green band Rrs; (**c**) Correlations between Rrs band ratio and in situ chlorophyll-a for VIIRS matching pairs and its corresponding in situ Rrs; (**d**) Correlations between VIIRS and in situ blue and green band Rrs; (**e**) Correlations between satellite estimated chlorophyll-a (VIIRS and MODIS, respectively) and in situ chlorophyll-a; (**f**) Correlations between various algorithms estimated chlorophyll-a and in situ chlorophyll-a. Algorithms include OC3M, OC3V, in situ Rrs band ratio algorithm (this manuscript), VIIRS Rrs band ratio algorithm (this manuscript).

We analyzed at sensor chlorophyll-a estimation from MODIS and VIIRS. Their chlorophyll-a products both show poor correlation with in situ chlorophyll-a (Figure 5e). Further comparisons between in situ and satellite blue or green bands display higher correlation from green band than blue band in both MODIS and VIIRS sensors (Figure 5b,d). However, VIIRS still performs better than MODIS in AP, in which both chlorophyll-a (Figure 5a,c) and Rrs (Figure 5b,d estimation are two times higher than MODIS.

The band set implies a potential improvement on chlorophyll-a estimation can be obtained if we had an ideal atmospheric correction. Current band ratio algorithms from field samples agree with chlorophyll-a within about 40% in MODIS and 50% in VIIRS (Figure 5a,c).

It is worth noticing that through OC3M and OC3V coefficients and algorithm constraints, their chlorophyll-a estimation increases from a low correlation (8.54% in MODIS and 9.91% in VIIRS, from Figure 5a,c) to the correlation of 17% in MODIS and 35% in VIIRS (Figure 5e).

Finally, we studied the coefficients for both OC3M and OC3V algorithms. Current algorithms appear to underestimate in >2 mg/m^3^ chlorophyll-a and overestimation in <2 mg/m^3^ chlorophyll-a samples (Figure 5f). No big difference exist between those two algorithms. Through all the comparisons, we improve the coefficients for VIIRS and increase its fitting coefficient to 53.12% (Table 2).

## 4. Discussion

### 4.1. Atmospheric Factors on Chlorophyll-a Retrieval

Since the atmospheric reflection signals occupy 90% of total ocean color satellite signal [25], atmospheric calibration becomes a significant part in global water ocean color products estimation. Scientific researchers need an accuracy of ~0.1% on sensor calibration, because 0.5% error in atmospheric correction will lead to a possible of ~5% mistake in processing nLw [29]. According to Mao et al. [30], visible bands show more sensitivity than near infrared bands in aerosol scatter radiance. Therefore, near infrared bands were suggested to be used as the reference band for atmosphere calibration, like 750 nm in open ocean [23] or higher bands in coastal waters [31]. In this study, we applied in situ skylight radiance to remove atmosphere signals and took 946 nm as the reference band for atmospheric correction. Thus, these in situ Rrs spectrums could be thought as undergoing ideal atmospheric correction. Its difference with satellite Rrs uncovers the deficiency of current satellite processing procedure on AP water.

Our research finds that several minutes delay in measurement leads to big gaps in skylight across visible bands (Figure 2 Skylight 1 & 2), though in an identical location. Because aerosol and gas distribution in atmosphere occur across quite larger spatial and temporal scales, the spectrum shape gap between Skylight 1 and Skylight 2 (Figure 2) can be caused by cloud contamination. Records from Kider et al. [24] reported similar signs when using an ASD for measuring clouds. Currently, research seldom focuses on the clouds’ impact on water-leaving reflectance, because most research usually removes cloud contaminated pixels directly. However, NASA pre-processing applies the NIR (near infrared) band (869 nm for MODIS, 862 nm for VIIRS) to avoid cloud contamination. Our result implies that clouds sometimes only have impacts on visible bands in AP (Skylight 2 in Figure 2), so the criteria flag on NIR band won’t be contaminated. And, NIR band limitation works better on cirrus detection. Cumulus are also prevalent in AP because of its low pressure, and marine/biological cloudy weather [11,32], which is hardly detected in the NIR bands. Actually, Martin et al. [33] applied 555 nm for cloud detection due to highly spatial variation of cloud. Therefore, standard protocol on ocean color products keeps those cloud contaminated pixels in images and increases AP Rrs uncertainty in visible bands, especially in green band. Green band of 555 nm is located right in the middle of atmosphere contaminated bands (Skylight 2), which have lower correlation with in situ Rrs in both MODIS and VIIRS (Figure 5b,d).

Current atmospheric simulation in SO reveals atmosphere model deficiency on cloud, aerosol and ozone simulation [34], e.g., the N_2_O (604 nm) and O_2_ (630 nm) gas absorption windows (red arrows in Figure 2). Therefore, we need more information and datasets to understand special SO atmosphere’s impact on its calibration from Rayleigh scatter, aerosol, ocean roughness and ocean bidirectional reflectance distribution [35,36]. Program SOCRATES pronounced great uncertainty and bias of cloud, aerosol and sea-air interaction simulation in SO, and hosted a funding research to improve atmospheric correction algorithms in 2016–2019 [37].

### 4.2. Bio-Optical Factors on Chlorophyll-a Retrieval

Lower water leaving reflectance values and narrower blue-green band ratios in AP than case I water shows different water optical characteristics between AP and global ocean water (Figure 3). The narrow band ratio changing rate in AP will have overestimation in low chlorophyll-a water and have underestimation in high chlorophyll-a water using the OC3 model. That is also the case shown by Figure 5f. Other reports show same satellite chlorophyll-a estimation issues in SO [38,39]. Therefore, OC-like empirical algorithm should be recalibrated by SO in situ data as suggested by Gracia et al. [40].

Research found a broad variety of special phytoplankton community live in AP, such as diatoms and phaeocystis spp [9]. Those diatoms have larger cell sizes [41] than in other places and also have unique absorption and scattering features for light regulating. Larger phytoplankton will increase the package effect and decrease the light/absorption efficiency, thus modulating different Rrs pattern [42].

Other water constituents in water will also decrease Rrs correlation relationship with phytoplankton and chlorophyll-a concentration. Field samples show high CDOM concentration around AP, which contributes 70% absorption of non-water absorption [43]. The main source of CDOM is from bacteria and krill in Weddell Sea and Bellingshausen Sea [44]. Those CDOM will interfere with the chlorophyll-a absorption peak [45], and cause uncertainty in chlorophyll-a estimation. In some cases, CDOM can be estimated by chlorophyll-a concentration [6,46]. Therefore, separating CDOM signal from Rrs (like bio-optical algorithm, GSM01 [47] and QAA [48]) is a better way to improve chlorophyll-a estimation in AP, where water is not only controlled by phytoplankton.

### 4.3. Sensor Factors on Chlorophyll-a Retrieval

Though with peculiar bio-optical properties, in situ Rrs from AP gets reliable chlorophyll-a estimations from an OC-like algorithm (Table 2 and Figure 5f). Those algorithms estimated chlorophyll-a range from 0.1 to 10 mg/m^3^, which have the same range with in situ chlorophyll-a. But there remains obvious underestimation in >2.0 mg/m^3^ chlorophyll-a and overestimation in <2.0 mg/m^3^ chlorophyll-a and their RMS surpasses 60% for both OC3M and OC3V algorithms. The estimation errors implied great deficiencies in OC-like algorithms’ coefficients. We improved the coefficients for OC3V and decreased its RMS to 29.97% and increased its chlorophyll-a estimation to a fitting coefficient of 53.12%. Those coefficients modified VIIRS regional chlorophyll-a estimation accuracy, though this regional algorithm still needs more validation. Other research on modifying OC-like regional coefficients in SO are mainly based on MODIS [11], and no regional coefficients modifications are for VIIRS as far as we knew.

Large gaps exist in satellite estimated chlorophyll-a and in situ chlorophyll-a (Figure 5e). Satellite estimated chlorophyll-a showed big underestimation for in situ chlorophyll-a (0.1–10 mg/m^3^), between 0.01–1 mg/m^3^. Other than coefficients, OC-like algorithm has another uncertainty source, band ratios. Sensitivity test (Figure 4) declares little difference in band ratio selection for both MODIS and VIIRS. But our results presented a better validation from VIIRS blue and green band Rrs measurements than MODIS (Figure 5b,d). Those two sensors (MODIS AQUA and VIIRS) have quite similar overpass times in the afternoon from 2 to 5 pm local time. Therefore, slight errors come from their measuring time gaps. The lower SNR, greater spectral range (Table 1) and higher spatial resolution (750 m) of VIIRS should be the reasons for its higher accuracy than MODIS (spatial resolution of 1 km in blue and green band) in AP. The broader coverage of VIIRS is also benefited from those sensor parameters. VIIRS gets its reprocessing improvement from its new SDR (Sensor data records) table and MSL12 (multi-sensor level-1 to level-2) data processing after 2014, which ocean color products are proved to be comparable to and even better than MODIS [49].

It is noted that VIIRS still needs more validation dataset for its products’ evaluation after its launch in 2011. SeaBASS dataset shows that current validation dataset for VIIRS doesn’t involve SO, especially >60° area [12]. Its better agreement with field samples are mostly concentrated on Hawaii, South Pacific Gyre, the U.S. East Coast, and the Gulf of Mexico coastal site [49]. Our data presented here proved its better application in AP chlorophyll-a estimation.

## 5. Conclusions

In this study, we collected hyper-spectral downwelling radiances and water-leaving Rrs around AP in January, 2014 and 2016. This hyper-spectral water bio-optical dataset helped to evaluate error sources from OC-like estimated chlorophyll-a in AP from three different aspects.

(1) Atmospheric correction. Though still lacking some absorption peaks in N_2_O (604 nm) and O_2_ (630 nm) absorption peaks, Mid-Latitude Summer simulation model shows the best agreement with field skylight downwelling radiance from current atmosphere simulation models. Besides, the cloud in AP appears a severe issue for satellite sensor detection. Because low amount of cumulus doesn’t cause absorption peaks in NIR band and only have impacts on visible green bands, satellite default cloud detection algorithm won’t remove those pixels from current NIR threshold algorithms. Thus, green bands Rrs show poor agreement with in situ water leaving Rrs than blue bands from both MODIS and VIIRS.
(2) Water bio-optics. The peculiar water bio-optical features in AP creates narrower band-ratio variability than global case I water, which enhances its bias in chlorophyll-a estimation, causing underestimation in high chlorophyll-a water and overestimation in low chlorophyll-a water.
(3) Sensor comparisons. VIIRS shows better performance than MODIS in detecting Rrs with an average of 74.522% accuracy in field: satellite correlations. VIIRS also has higher spatial coverage than MODIS. After coefficient improvement, its chlorophyll-a estimation could reach a fitting coefficient of 53.12% and a RMS of 29.97%.

Our hyper-spectral data from AP build a new way in understanding ocean color algorithms and provide an improved validation dataset for the newly launch VIIRS sensor. This study also shows a promising future for VIIRS ocean color products application in SO area.

## Figures and Tables

**Table 1:** Matching pairs statistics for MODIS and VIIRS.

Satellite	Matching Probability (Total In Situ = 39)	Valid Probability ^1^ (%)	Band	Average of Std ^2^	Correlation Coefficient	SNR ^3^	Band Width (nm)
MODIS	61.54% (matching pairs = 24)	4.9 ± 1.7	412	0.0071	−9.21%	1208	15
			443	0.0079	−12.39%	1325	15
			469 (0.5 km)	0.0088	−20.64%	316	20
			488	0.0093	−30.31%	1308	10
			531	0.0114	−28.67%	1385	10
			547	0.0122	−20.01%	1114	10
			555 (0.5 km)	0.0129	−25.88%	302	20
			645 (0.25 km)	0.0040	−3.17%	168	50
			667	0.0031	42.10%	1163	10
			678	0.0028	37.94%	1265	10
VIIRS	76.92% (matching pairs = 30)	6.0 ± 1.2	410	0.0064	74.37%	827	20
			443	0.0090	72.67%	774	18
			486	0.0101	68.54%	747	20
			551	0.0126	78.58%	586	20
			671	0.0032	78.45%	450	20

^1^ statistical daily average in whole Jan, 2014 for SO higher than 60S ocean; ^2^ standard deviation; ^3^ signal noise ratio.

**Table 2:** Band ratio algorithms and their coefficients used in our study.

Band Ratio Algorithm							
$\text{chl}=10^{a0+a1\times x+a2\times x^{2}+a3\times x^{3}+a4\times x^{4}}$, $x=\frac{\text{max}\left(blue\;band\right)}{green\;band}$							
	a0	a1	a2	a3	a4	r2	RMSE *
OC3M	0.2424	−2.7423	1.8017	0.0015	−1.228	50.85%	0.6304
OC3V	0.2228	−2.4683	1.5867	−0.5275	−0.7768	51.44%	0.6688
In situ Rrs band ratio algorithm (this manuscript)	0.3722	−7.74	−3.876	269.9	−261.9	62.38%	0.2762
VIIRS Rrs band ratio algorithm (this manuscript)	−4.177	31.85	4.1	−297.1	383.6	53.12%	0.2997

* RMSE is $\frac{\sqrt[{2}]{\sum \left(\left(\text{estimated chla}\right)-\left(\text{field chla}\right)\right)2}}{N}$.
